# RETRACTED: Amin et al. Liver Tumor Localization Based on YOLOv3 and 3D-Semantic Segmentation Using Deep Neural Networks. *Diagnostics* 2022, *12*, 823

**DOI:** 10.3390/diagnostics16172728

**Published:** 2026-08-26

**Authors:** Javaria Amin, Muhammad Almas Anjum, Muhammad Sharif, Seifedine Kadry, Ahmed Nadeem, Sheikh F. Ahmad

**Affiliations:** 1 Department of Computer Science, University of Wah, Wah Cantt 47040, Pakistan; javeria.amin@uow.edu.pk; 2 National University of Technology (NUTECH), Islamabad 44000, Pakistan; almasanjum@yahoo.com; 3 Department of Computer Science, Comsats University Islamabad, Wah Campus, Wah Cantt 47040, Pakistan; muhammadsharifmalik@yahoo.com; 4 Department of Applied Data Science, Noroff University College, 4609 Kristiansand, Norway; 5 Department of Pharmacology & Toxicology, College of Pharmacy, King Saud University, P.O. Box 2455, Riyadh 11451, Saudi Arabia; anadeem@ksu.edu.sa (A.N.); fashaikh@ksu.edu.sa (S.F.A.)

*Diagnostics* retracts the article entitled “Liver Tumor Localization Based on YOLOv3 and 3D-Semantic Segmentation Using Deep Neural Networks” [1] cited above.

Following publication, concerns were brought to the attention of the Editorial Office regarding the appropriateness of the reference list of this article [1].

In accordance with standard journal procedures, the Editorial Office and Editorial Board conducted an investigation that confirmed a significant number of inappropriate citations that lacked sufficient relevancy to the context in which they were presented. While the authors cooperated with the investigation, their responses did not satisfactorily address these concerns. As a result, the Editorial Board has lost confidence in the integrity of the article and has therefore decided to retract this publication [1], in accordance with MDPI’s retraction policy.

This retraction was approved by the Editor-in-Chief of *Diagnostics*.

The authors have been informed of this retraction and have indicated their disagreement with this decision.
